# Sociospatial patterning of the use of new transport infrastructure: Walking, cycling and bus travel on the Cambridgeshire guided busway

**DOI:** 10.1016/j.jth.2014.10.006

**Published:** 2015-06

**Authors:** Eva Heinen, Jenna Panter, Alice Dalton, Andy Jones, David Ogilvie

**Affiliations:** a MRC Epidemiology Unit and UKCRC Centre for Diet and Activity Research (CEDAR), University of Cambridge School of Clinical Medicine, Box 285, Cambridge Biomedical Campus, Cambridge CB2 0QQ, United Kingdom; b Norwich Medical School, University of East Anglia, Norwich Research Park, Norwich NR4 7TJ, United Kingdom

**Keywords:** Transport infrastructure, Walking, Cycling, Bus use, Intervention, Socioeconomic characteristics

## Abstract

**Background:**

New transport infrastructure may help promote active travel, thereby contributing to increasing overall physical activity and population health gain. In 2011 a guided busway with a path for walking and cycling was opened in Cambridgeshire, UK. This paper investigates the predictors of walking, cycling and bus use on the busway.

**Methods:**

Cross-sectional analyses of the final questionnaire wave (2012) of the Commuting and Health in Cambridge cohort study following the opening of the busway. Participants were 453 adult commuters who had not moved home or workplace. Busway use was self-reported and proximity calculated using GIS. Separate multivariable logistic regression models were used to assess predictors of walking, cycling and bus use on the busway.

**Results:**

Exposure to the intervention (proximity: the negative square root of the distance from home to busway in kilometres) increased the odds of use for cycling (OR 2.18; 95% CI 1.58 to 3.00), bus travel (OR 1.53, 95% CI 1.15 to 2.02) and walking (OR 1.34; 95% CI 1.05 to 1.70). The effect of exposure was strengthened in towns for bus use, and in towns and villages for walking, compared with urban areas. Men were more likely than women to have cycled on the busway, whereas individual socioeconomic characteristics did not predict bus use or walking.

**Conclusion:**

New high-quality transport infrastructure attracts users, determined by geographical exposure and spatial contextual factors such as settlement size and availability of parking at work. Future longitudinal analyses will determine effects on overall travel and physical activity behaviour change.

## Highlights

• New infrastructure may promote active travel and thereby contribute to health gain.
• A new guided busway and service path attracted walkers, cyclists and bus users.
• Use was determined by residential proximity to the intervention.
• Proximity was a stronger predictor of use for cycling than for walking or bus use.
• The effect of proximity was stronger in the population living outside urban areas.

## 1 Introduction

The prospect of improving health by increasing physical activity has obtained worldwide attention (United Nation General Assembly, 2011). Active travel – walking and cycling for transport – offers an easily integrated form of everyday physical activity that can be sufficient to improve health and well-being (Chief Medical Officers, 2011). However, not all environments are equally supportive for walking and cycling (Handy et al., 2002; Saelaens and Handy, 2008; Titze et al., 2008). New infrastructure could contribute to an increase in active travel, either by inducing additional walking or cycling trips or by shifting existing trips to these modes of travel. Improving public transport could also help achieve this, because public transport use is associated with higher levels of active travel **(**Rissell et al., 2012; Freeland et al., 2013; Lachapelle and Noland**,** 2012; Besser and Dannenberg, 2005).

Conventional wisdom suggests that providing safe and high-quality infrastructure may attract users, and greater provision of bicycle infrastructure has indeed been associated with higher levels of cycling (Dill and Carr, 2003). People may be willing to detour to use a facility. For example, Dill (2009) showed that cyclists travelled a disproportionate share of their journeys on dedicated infrastructure. On the other hand, proximity to infrastructure appears to increase its use. For example, Krizek et al. (2007) demonstrated that urban cycle path use declined with increasing distance from home to path. Similarly, whether people walk or cycle to a bus stop or railway station is associated with the distance involved (Martens, 2004). However, evidence for any maximum acceptable access distance is inconclusive (Guerra et al., 2011), and although the distance decay function appears to differ between modes of travel (Iacono et al., 2008) it is unknown if this also applies to the use of different modes on the same infrastructure.

Few intervention studies have provided causal evidence linking changes in the built environment with changes in active travel (Ogilvie et al., 2007; Yang et al., 2010). However, Goodman et al. (2013), (2014) have recently shown in a longitudinal quasi-experimental study that proximity to new walking and cycling routes predicted their use, and increases in overall physical activity, among residents living within 5 km. The number of destinations that can be reached may be another important predictor of use. While such accessibility benefits are likely to differ between individuals as a result of different spatial activity patterns, there is little evidence that individuals expected to benefit more from new infrastructure use it more (Chatterjee and Ma, 2009). The few intervention studies available have tended to focus on single modes of transport and have also often ignored the social and geographical patterning of exposure and response to new transport infrastructure, which is important for evaluating its population health impacts.

The Commuting and Health in Cambridge study offers the opportunity to explore the extent to which exposure to new infrastructure is associated with changes in travel behaviour using that infrastructure. In 2011 a guided busway with a path for walking and cycling was opened in and around Cambridge, UK (Ogilvie et al., 2010). This construction — the longest of its kind in the world – is unique in providing facilities for three modes of transport in one intervention, including a bus service of higher than usual quality for the UK. An ethnographic study has provided insight into the factors that early users of the busway found important and how it may have changed their travel behaviour (Jones et al., 2013). However, it remains unclear which factors contribute to the use of different modes of travel on the busway. The aim of this paper is to test the first step of a putative causal pathway linking this transport intervention with population health impacts, by determining the relationship between the provision of new infrastructure and its use.

## 2 Methods

### 2.1 The Cambridgeshire guided busway

**Fig. 1:**
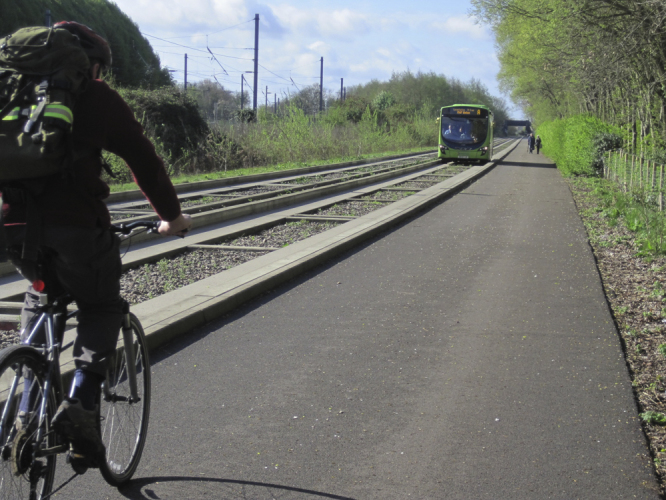
The Cambridgeshire guided busway

**Fig. 2:**
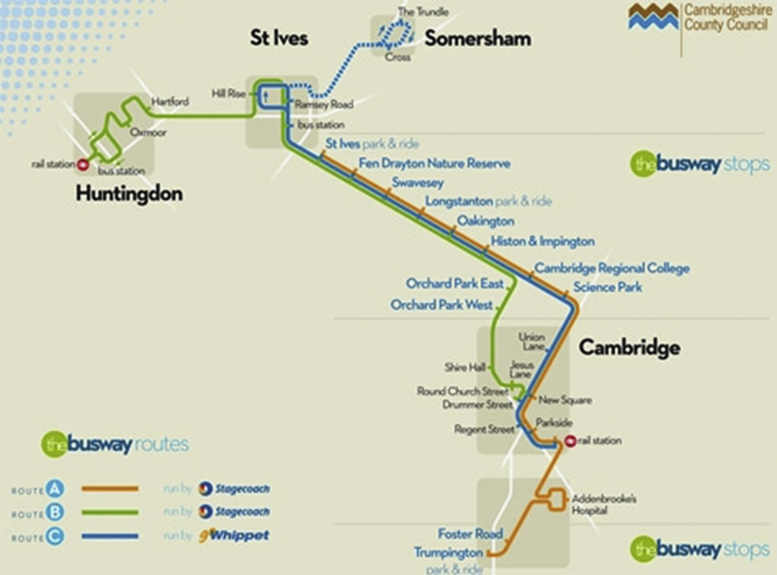
Map of the Cambridgeshire Guided Busway (Map reproduced with kind permission of Cambridgeshire County Council)

The study is set in and around the city of Cambridge, UK (123,900 inhabitants) (ONS, 2011). The busway was opened in August 2011. It consists of a 25 km guideway (separate off-road track) for specially adapted buses, accompanied by a service path that can be used for walking and cycling (Figs. 1 and 2). The busway links several major employment sites in the city centre and urban fringes with outlying towns and villages, and includes new park-and-ride facilities at each terminus and at a third, rural stop at Longstanton (www.thebusway.info).

### 2.2 Study sample

Data were collected as part of a longitudinal cohort study of adults aged 16 years or over working in areas of Cambridge to be served by the busway, living within a radius of approximately 30 km of the city centre, and recruited predominantly through workplaces (Ogilvie et al., 2010; Panter et al., 2011). The fourth and final (post-intervention) annual wave of survey data was collected by post from the 665 remaining cohort participants in 2012. This survey was approved by the University of Cambridge Psychology Research Ethics Committee (reference no. 2012.14) and all participants provided written informed consent. For the present analysis we excluded the 212 respondents who had moved home or workplace at any time during the cohort study (2009–2012), because people who have recently moved are more open to reconsidering their travel behaviour (Verplanken et al., 2008; Bamberg, 2006) and moving could therefore have influenced busway use in a variety of ways that could not (easily) be controlled for. This left 453 participants included in the analyses.

### 2.3 Outcome measures

Three binary dependent variables were used: use of the guided bus, use of the path for cycling and use of the path for walking. These were ascertained using the questions ‘Have you ever travelled on a guided bus in Cambridgeshire?’ (response options: ‘Yes’ or ‘No’) and ‘Have you ever walked or cycled along any part of the footpath or cycle path beside the guided busway?’ (response options, of which more than one could be selected: ‘Yes – I have walked beside the busway’, ‘Yes – I have cycled beside the busway’ and/or ‘No – I have not walked or cycled along the path beside the busway at all’).

Walking and cycling had been possible on the path before its official opening. If participants had indicated using the path in wave two (2010) or three (2011) of the survey but not in wave four (2012) (walking, *n*=35; cycling, *n*=12), we carried forward these positive responses to wave four. Sensitivity analysis showed that restoring these imputed positive responses to their original values made no substantial difference to the results.

Almost all participants (99.1%) knew of the busway; 30.7% reported having used the guided bus, 40.0% having cycled on the path and 32.2% having walked on the path

### 2.4 Exposure measures

Different measures of exposure to the intervention were used for the analyses of different outcomes (Appendix 1). For guided bus use, exposure was defined using the proximity of each participant’s home to the nearest busway stop and the modelled change in bus travel time to work induced by the intervention. For walking and cycling, exposure was defined using proximity to the nearest access point to the path and the change in the modelled walking/cycling distance to work induced by the intervention.

The distance to the nearest busway stop or path access point was the primary measure of accessibility of the intervention in general. We expected a given increment in distance to have a smaller effect on use as distance increased, which suggested that it would not be appropriate to model a linear relationship. Exploratory analyses confirmed this. Whilst the log transform is also commonly used in studies of distance decay, the root square transformation was selected as the estimates produced are slightly more conservative and more easily interpretable. For ease of interpretation, *proximity* to the intervention — the inverse (i.e. the negative) of the square root of the distance — was used as the primary exposure measure in all models.

We were also able to estimate the changes in travel time or distance to work for each participant attributable to the intervention. Because the outcome measures captured use of the intervention for any purpose, these commuting-specific metrics were used as secondary exposure measures to indicate the degree to which an individual participant might benefit in respect of their journey to work, and to serve as proxies for potential benefits in respect of other trips.

### 2.5 Covariates

The following covariates, ascertained using questionnaire items reported previously (Panter et al., 2011), were included in analysis: gender, age, education level, car ownership, housing tenure, possession of a driving licence, access to a bicycle, being a student, presence of children in the household, presence of a limiting long-term health condition, difficulty walking, and the mental (MCS-8) and physical (PCS-8) summary scores of the SF-8 (Ware et al., 2008) along with an indicator of residential settlement size – the urban/rural classification of the census output area of each participant׳s home postcode (Bibby and Shephard, 2004) (Table 1). Because participants were all commuters, the length of their shortest route on the pedestrian and cyclist network from home to work (before the intervention) and self-reported availability of (free) parking at work – both of which have been shown to be associated with mode of travel to work (Heinen et al., 2013) – were also included as covariates. Any missing values for covariates were substituted by the last reported value in previous survey waves (limiting long-term health conditions, *n*=4; difficulty walking, *n*=2). Sensitivity analyses showed no effect of this substitution.

### 2.6 Analysis

Separate multivariable logistic regression models were estimated for each of the three outcomes of guided bus use and walking and cycling on the path. Gender, age and any other explanatory variables associated with the outcome at *p*<0.25 in unadjusted models were included in the multivariable models. Interaction effects were tested between proximity to the intervention (primary exposure measure) and gender, age, settlement size, and the modelled change in travel time or distance to work (secondary exposure measure). Three versions of each multivariable model were estimated: a maximally adjusted model including interaction effects; a model without interaction effects; and a model excluding workplace characteristics. In the final models, the distance from home to work was omitted because it was highly correlated with the primary exposure measure in each model (*r*=0.75 for the bus model, *r*=0.83 for the walking and cycling models). The numbers of individuals who were students or reported difficulties walking were small; these variables were therefore also omitted.

## 3 Results

### 3.1 Participant characteristics

Participants had a mean age of 47.6 years and 70% were women. While our sample had a high prevalence of car ownership comparable with that of the area from which they were drawn, women and graduates were over-represented in the sample and younger adults were under-represented (Appendix 2). Respondents lived on average around 7 km from the busway, with those in more urban settlements tending to live closer. 86 (19.5%) participants were estimated to have experienced a change in walking/cycling distance to work following the intervention, and 46 (10.5%) a change in bus travel time to work (Table 1). These changes tended to be small, with median changes of −0.1 km and 4.0 min respectively among those experiencing any change at all.

### 3.2 Predictors of guided bus use

Living closer to the busway was associated with an increased likelihood of using the guided bus (OR 1.53, 95% CI 1.15 to 2.02) (Table 2). This corresponds to a 53% increase in the odds of having used the guided bus for those living 4 km from the busway compared to those living 9 km away.

Compared to living in an urban area, living in a town or fringe location (OR 3.45, 95% CI 1.62 to 7.37) or in a village, hamlet or isolated dwelling (OR 2.09, 95% CI 1.04 to 4.21) increased the odds of having used the guided bus. Using the bus was not associated with any of the individual socioeconomic characteristics investigated.

One significant interaction effect was identified: the effect of proximity to the busway was strengthened among those living in town and urban fringe locations (OR 2.95, 95% CI 1.42 to 6.15).

### 3.3 Predictors of walking on the path

Proximity to the busway was associated with having walked on the path (OR 1.34, 95% CI 1.05 to 1.70) (Table 3), which means that an individual living 4 compared to 9 km away from the busway has 34% higher odds to have walked on the busway. We also found an effect of an interaction term between proximity and settlement size. This interaction term predicted the likelihood of walking on the path, in that individuals living closer to the busway were more likely to walk on the path if they lived in towns and urban fringe locations (OR 2.91, 95% CI 1.52 to 5.55) or villages (OR 2.20, 95% CI 1.22 to 3.97).

Compared to having no parking, having free or paid car parking at work was associated with increased odds of walking on the path (respectively OR 2.29, 95% CI 1.29 to 4.06; OR 2.71, 95% CI 1.42 to 5.15). Excluding workplace characteristics from the multivariable model had only a small effect on the adjusted odds ratio for the main effect of the intervention, suggesting that the walking captured in this model included walking for commuting and other purposes.

### 3.4 Predictors of cycling on the path

Proximity to the busway was associated with use of the path for cycling (OR 2.18, 95% CI 1.58 to 3.00) (Table 4). An individual living 4 km from the path was more than twice as likely to have cycled on the path than one living 9 km away.

Unlike for guided bus use and walking, socioeconomic characteristics were associated with use of the path for cycling. Women were less likely than men to report having cycled (OR 0.41, 95% CI 0.24 to 0.69). People living in town or urban fringe locations (OR 2.10, 95% CI 1.04 to 4.21) or villages or hamlets (OR 2.65, 95% CI 1.21 to 5.78) were more likely to have cycled on the path. Workplace characteristics were not associated with use of the path for cycling, and no significant interaction terms were found.

## 4 Discussion and conclusion

The results indicate that the new high-quality infrastructure and public transport service provided by the Cambridgeshire Guided Busway attracted users, and that among a population of adult commuters its use was clearly associated with geographical exposure in terms of residential proximity to the busway.

The association of use with proximity was stronger for cycling than for walking or bus use (unadjusted ORs: bicycle 2.30; bus 1.54; walk 1.63). Krygsman et al. (2004) suggest that the time for which people are willing to travel to access a public transport service increases with the total journey duration. Guided bus users may have been likely to travel further than cyclists on each trip, and therefore less sensitive to a given increment in access distance. Walking on the path as captured in the survey could reflect a mixture of walking to access the bus service and walking in its own right, which may explain an odds ratio for walking that was intermediate between those for bus use and for cycling. Secondary measures of exposure to the intervention in terms of its effect in reducing distance or travel time to work did not predict use in multivariable models, perhaps reflecting the small absolute changes in these measures.

Spatial contextual factors, such as settlement size and the availability of car parking at work, were also associated with the use of the busway by all three modes of transport. The effect of proximity to the intervention was strengthened in towns for bus use, and in towns and villages for walking, compared with urban areas. These findings indicate that the uptake of the intervention was moderated by characteristics of the settlements in which people lived, suggesting that its ultimate effects may not be equally distributed across the area it serves.

Significant associations between use of the intervention and socioeconomic characteristics were observed only for cycling: we found that men were more likely than women to cycle on the busway, corresponding with the social patterning of cycling in the UK in general (DfT, 2013). We did not find evidence of a socioeconomic patterning of guided bus use. This suggests that the guided bus service may have been regarded as more acceptable to higher socioeconomic groups than ordinary bus services, which may be important for the normalisation and destigmatisation of bus travel as an alternative to car travel (Jones et al., 2013).

The key strengths of this study lie in its use of measures of individual exposure to show that new transport infrastructure attracts users, and in its exploration of how that use is socioeconomically patterned and moderated by other characteristics of the built environment. In doing so, we have confirmed the first step of a putative causal pathway linking this novel environmental intervention with potential impacts on population activity patterns and health. Nevertheless, the study has a number of limitations. These include its focus on a sample of commuters not entirely representative of the population of Cambridgeshire – who may use the busway more or less frequently, in different ways and for different reasons than the general population – and its reliance on a self-reported measure of infrastructure use, the validity of which may be threatened by intentional or unintentional misreporting. Furthermore, the analyses were not specific to a particular journey purpose, whereas use of the busway may have been associated with different determinants for different trip purposes. Data collected in a separate, larger intercept survey of busway users may provide additional insight into the characteristics of use, such as trip purpose and frequency.

Despite its limitations, the findings of this study are important for public health for two reasons. First, we have shown that people will take up the opportunity to walk, and particularly to cycle, on high quality infrastructure, even when high quality public transport is also provided. This suggests that public transport can coexist with active travel in a more sustainable and health-promoting transport system, rather than necessarily deterring people from walking or cycling (Edwards et al., 2013). Second, we have shown that the uptake of such an intervention may be greater in the population living outside urban areas, where there may be more potential for shifting away from the private car for everyday mobility. While the findings suggest that the busway could result in higher levels of active travel and consequent health improvement, the next steps in the causal pathway remain to be tested. Future longitudinal analyses of the cohort data will aim to quantify and understand changes in travel behaviour (modal shift, trip frequency or distance travelled) and overall activity patterns (including any compensatory decrease in other domains of physical activity) to determine the extent to which the intervention has resulted in changes sufficient to improve physical activity and public health.

## Authors׳ contributions

EH and DO conceived of the study and wrote the manuscript. EH conducted the analysis and drafted the manuscript. AD and AJ calculated the objective spatial measures. JP, AD and AJ advised on the design of the study and the interpretation of the emerging findings and contributed to the critical revision of the paper. All authors read and approved the final manuscript.

## Figures and Tables

**Table B1:** Comparison of characteristics of study population with local resident population

**Characteristic**	**Prevalence in study sample (%)**	**Prevalence in local resident population aged aged 16–64 (%)**	**Reference population**
**Demographic**			
Female	70.8	48	Cambridge city
		50	South Cambridgeshire
		50	East Cambridgeshire
Aged 16–30	4.6	43	Cambridge city
		16	South Cambridgeshire
		15	East Cambridgeshire
**Socio-economic**			
Degree-level or equivalent education*	64.1	50	Cambridge city
		44	South Cambridgeshire
		32	East Cambridgeshire
Access to a car*	90.2	73	Cambridge city
		95	South Cambridgeshire
		94	East Cambridgeshire

⁎ Aged 16–64. Source: Census (2011) Standard Area Statistics (England and Wales) (http://www.neighbourhood.statistics.gov.uk). The locsal authority areas of South Cambridgeshire and East Cambridgeshire represent the majority of the surrounding rural area from which the cohort was drawn.

**Table 1:** Summary of participant characteristics

		*n*	%	mean	st.d.	total *n*
Used the guided bus	No	310	68.4			449
	Yes	139	30.7			
Used the path for cycling	No	272	60.0			453
	Yes	181	40.0			
Used the path for walking	No	307	67.8			453
	Yes	146	32.2			
Distance to guided bus stop (km)				6.90	8.12	440
Proximity to stop (negative square root of distance in km)				−2.21	1.42	440
Distance to path (km)				7.23	8.01	440
Proximity to path (negative square root of distance in km)				−2.31	1.38	440
Change in commute distance (km)				−0.05	0.19	440
Change in commute travel time by public transport (_min_)				−0.84	4.01	428
Gender	Male	128	29.2			438
	Female	310	70.8			
Age at t4				47.6	11.1	438
Age in categories	≤30	20	4.6			438
	31–40	106	24.2			
	41–50	116	26.5			
	51–60	136	31.1			
	61+	60	13.7			
Education level	Degree	280	64.1			437
	Less than degree	157	35.9			
Car ownership	No	44	9.8			450
	One car	214	47.6			
	Two or more cars	192	42.7			
Housing tenure	Not owner	76	16.8			453
	Owner	377	83.2			
Driving licence	No	38	8.4			451
	Yes	413	91.6			
Access to a bicycle	No	61	13.5			451
	Yes	390	86.5			
Student	No	445	98.2			453
	Yes	8	1.8			
Children in household	No	330	72.9			453
	Yes	123	27.2			
Mental health (MCS-8)				51.0	9.4	453
Physical health (PCS-8)				52.9	8.9	453
Limiting health condition	No	402	88.7			453
	Yes	51	11.3			
Difficulty walking	No	441	97.4			453
	Yes	12	2.7			
Type of settlement	Urban (>10,000)	280	64.1			437
	Town & Fringe	78	17.9			
	Village, Hamlet & Isolated Dwellings	79	18.1			
Car parking at work	No	115	25.4			452
	Yes, paid	124	27.4			
	Yes, free	213	47.1			
Commute distance (km)				11.44	9.50	440

**Table 2:** Predictors of guided bus use

Variable	Unadjusted		Maximally adjusted		Adjusted model without interaction effects		Adjusted model without workplace characteristics	
	OR (95% CI)	*p*	OR (95% CI)	*p*	OR (95% CI)	*p*	OR (95% CI)	*p*
Intervention								
Proximity to stop (negative square root of distance in km)	**1.54 (1.29, 1.82)**	**<0.001**	**1.53 (1.15, 2.02)**	**0.003**	**1.89 (1.47, 2.41)**	**<0.001**	**1.53 (1.16, 2.02)**	**0.003**
Change in commute travel time by public transport (_min_)	**0.91 (0.85, 0.97)**	**0.004**	1.03 (0.96, 1.11)	0.439	1.06 (0.99, 1.14)	0.071	1.03 (0.96, 1.11)	0.421
Socioeconomic characteristics								
Gender								
Male	1.00		1.00		1.00		1.00	
Female	1.29 (0.82, 2.03)	0.276	1.39 (0.81, 2.40)	0.233	1.34 (0.79, 2.27)	0.281	1.48 (0.86, 2.53)	0.156
Age								
≤30	0.58 (0.38, 3.09)	0.877	1.21 (0.37, 3.94)	0.746	1.15 (0.36, 3.60)	0.815	1.19 (0.37, 3.83)	0.767
31–40	1.00		1.00		1.00		1.00	
41–50	1.12 (0.63, 2.01)	0.697	1.18 (0.61, 2.30)	0.618	1.11 (0.58, 2.14)	0.748	1.22 (0.63, 2.36)	0.553
51–60	1.28 (0.74, 2.23)	0.381	1.27 (0.66, 2.45)	0.481	1.31 (0.69, 2.49)	0.412	1.31 (0.68, 2.51)	0.425
61+	1.36 (0.69, 2.69)	0.370	1.38 (0.60, 3.17)	0.443	1.32 (0.59, 2.97)	0.495	1.49 (0.66, 3.37)	0.342
Age								
Years	1.01 (0.99, 1.03)	0.433						
Education level								
No degree	1.00							
Degree	0.95 (0.63, 1.46)	0.829						
Car ownership								
No	1.00		1.00		1.00		1.00	
1 car	0.75 (0.38, 1.47)	0.401	0.61 (0.28, 1.34)	0.218	0.63 (0.29, 1.36)	0.236	0.61 (0.28, 1.31)	0.204
2 or more	0.61 (0.31, 1.21)	0.157	0.56 (0.24, 1.31)	0.184	0.63 (0.27, 1.44)	0.272	0.53 (0.23, 1.22)	0.136
Housing tenure								
Not owner	1.00							
Owner	0.94 (0.55, 1.61)	0.831						
Driving licence								
No	1.00							
Yes	0.75 (0.38, 1.50)	0.419						
Access to bicycle								
No	1.00							
Yes	0.84 (0.47, 1.48)	0.537						
Student								
No	1.00							
Yes	0.89 (0.17, 4.65)	0.891						
Child(ren) in household								
No	1.00							
Yes	1.05 (0.67, 1.64)	0.844						
Health characteristics								
Mental health								
(MCS-8)	1.02 (0.99, 1.05)	0.110	1.03 (0.99, 1.06)	0.129	1.03 (0.99, 1.06)	0.104	1.02 (0.99, 1.06)	0.143
Physical health								
(PCS-8)	1.01 (0.98, 1.04)	0.456						
Limiting health condition								
No	1.00		1.00		1.00		1.00	
Yes	0.66 (0.33, 1.30)	0.226	0.65 (0.30, 1.43)	0.289	0.67 (0.31, 1.41)	0.290	0.66 (0.30, 1.44)	0.296
Difficulty walking								
No	1.00							
Yes	0.44 (0.09, 2.03)	0.291						
Spatial characteristics								
Type of settlement								
Urban	1.00		1.00		1.00		1.00	
Town & Fringe	**1.84 (1.09, 3.09)**	**0.022**	**3.45 (1.62, 7.37)**	**0.001**	**3.31 (1.67, 6.58)**	**0.001**	**3.39 (1.61, 7.14)**	**0.001**
Village, Hamlet & Isolated Dwellings	1.11 (0.64, 1.92)	0.699	**2.09 (1.04, 4.21)**	**0.038**	**2.40 (1.18, 4.85)**	**0.015**	**2.01 (1.00, 4.03)**	**0.049**
Work characteristics								
Car parking at work								
No	1.00		1.00		1.00			
Yes, paid	0.88 (0.51, 1.50)	0.635	0.93 (0.50, 1.75)	0.824	0.91 (0.49, 1.68)	0.755		
Yes, free	0.65 (0.40, 1.05)	0.080	0.59 (0.33, 1.04)	0.067	0.60 (0.34, 1.05)	0.075		
Commute distance (km)	1.01 (0.99, 1.03)	0.442						
Interaction variables								
Proximity *Change in travel time			1.13 (1.00, 1.27)	0.055			1.12 (0.99, 1.26)	0.072
								
Proximity * Town & Fringe			**2.95 (1.42, 6.15)**	**0.004**			**2.94 (1.41, 6.11)**	**0.004**
Proximity * Village, Hamlet & Isolated Dwellings			1.29 (0.74, 2.24)	0.369			1.25 (0.72, 2.15)	0.424
Constant			0.30 (0.04, 2.09)	0.223	0.37 (0.06, 2.39)	0.296	0.23 (0.03, 1.60)	0.138
			n=412		n=412		n=413	

**Table 3:** Predictors of walking on the path

Variable	Unadjusted		Maximally adjusted		Adjusted model without interaction effects		Adjusted model without workplace characteristics		Sensitivity analysis: use not coded forward	
	OR (95% CI)	p	OR (95% CI)	p	OR (95% CI)	p	OR (95% CI)	p	OR (95% CI)	p
Intervention										
Proximity to path (negative square root of distance in km)	**1.63 (1.37, 1.95)**	**<0.001**	**1.34 (1.05, 1.70)**	**0.017**	**1.88 (1.50, 2.35)**	**<0.001**	**1.31 (1.04, 1.66)**	**0.023**	**2.00 (1.55, 2.58)**	**<0.001**
Change in commute distance (km)	0.65 (0.24, 1.72)	0.383								
Socio-economics										
Gender										
Male	1.00		1.00		1.00		1.00		1.00	
Female	0.84 (0.54, 1.29)	0.419	0.81 (0.49, 1.33)	0.398	0.82 (0.50, 1.33)	0.421	0.80 (0.49, 1.30)	0.36	0.93 (0.55, 1.58)	0.794
Age										
≤30	1.19 (0.45, 3.17)	0.724	1.46 (0.50, 4.25)	0.489	1.25 (0.44, 3.54)	0.678	1.63 (0.57, 4.63)	0.361	0.73 (0.22, 2.47)	0.613
31–40	1.00		1.00		1.00		1.00		1.00	
41–50	0.68 (0.39, 1.20)	0.187	0.65 (0.35, 1.23)	0.185	0.65 (0.35, 1.21)	0.178	0.67 (0.36, 1.24)	0.204	0.77 (0.40, 1.49)	0.439
51–60	1.04 (0.61, 1.76)	0.883	1.10 (0.61, 2.01)	0.749	1.11 (0.61, 2.00)	0.74	1.06 (0.59, 1.92)	0.835	1.19 (0.64, 2.21)	0.592
61+	0.71 (0.36, 1.41)	0.324	0.72 (0.33, 1.58)	0.412	0.70 (0.33, 1.51)	0.366	0.70 (0.32, 1.51)	0.363	0.45 (0.18, 1.10)	0.080
Age										
Years	0.99 (0.98, 1.01)	0.581								
Education level										
No degree	1.00									
Degree	1.15 (0.76, 1.75)	0.513								
Car ownership										
No	1.00		1.00		1.00		1.00		1.00	
1 car	0.96 (0.49, 1.89)	0.915	0.71 (0.33, 1.52)	0.38	0.74 (0.34, 1.60)	0.449	0.82 (0.39, 1.72)	0.603	0.93 (0.40, 2.16)	0.862
2 or more	0.65 (0.33, 1.30)	0.222	0.53 (0.24, 1.21)	0.134	0.58 (0.25, 1.32)	0.191	0.69 (0.32, 1.51)	0.355	0.84 (0.34, 2.08)	0.710
Housing tenure										
Not owner	1.00									
Owner	0.96 (0.57, 1.63)	0.892								
Driving licence										
No	1.00									
Yes	0.80 (0.40, 1.59)	0.518								
Access to bicycle										
No	1.00									
Yes	0.89 (0.50, 1.57)	0.683								
Student										
No	1.00									
Yes	2.13 (0.53, 8.65)	0.289								
Child(ren) in household										
No	1.00									
Yes	1.07 (0.69, 1.66)	0.759								
Health characteristics										
Mental health										
(MCS-8)	0.99 (0.97, 1.02)	0.495								
Physical health										
(PCS-8)	1.01 (0.98, 1.04)	0.489								
Limiting health condition										
No	1.00									
Yes	1.29 (0.70, 2.36)	0.416								
Difficulty walking										
No	1.00									
Yes	1.05 (0.31, 3.55)	0.934								
Spatial characteristics										
Type of settlement										
Urban	1.00		1.00		1.00		1.00		1.00	
Town & Fringe	1.11 (0.66, 1.87)	0.702	1.93 (0.99, 3.77)	0.055	**2.14 (1.15, 4.00)**	**0.017**	**1.97 (1.01, 3.84)**	**0.047**	**2.93 (1.51, 5.69)**	**0.001**
Village, Hamlet & Isolated Dwellings	0.81 (0.47, 1.40)	0.456	1.59 (0.81, 3.11)	0.174	1.68 (0.87, 3.25)	0.121	1.58 (0.81, 3.07)	0.176	**2.10 (1.03, 4.30)**	**0.043**
Work characteristics										
Car parking at work										
No	1.00		1.00		1.00				1.00	
Yes, paid	1.71 (0.97, 3.00)	0.062	**2.71 (1.42, 5.15)**	**0.002**	**2.50 (1.34, 4.67)**	**0.004**			1.88 (0.95, 3.71)	0.071
Yes, free	1.65 (0.99, 2.76)	0.053	**2.29 (1.29, 4.06)**	**0.005**	**2.19 (1.26, 3.83)**	**0.006**			**2.02 (1.11, 3.70)**	**0.022**
Commute distance (km)	**0.98 (0.96, 1.00)**	**0.036**								
Interaction variables										
										
Proximity * Town & Fringe			**2.91 (1.52, 5.55)**	**0.001**			**2.76 (1.46, 5.21)**	**0.002**		
										
Proximity * Village, Hamlet & Isolated Dwellings			**2.20 (1.22, 3.97)**	**0.009**			**2.08 (1.17, 3.68)**	**0.012**		
Constant			0.84 (0.32, 2.18)	0.722	1.46 (0.57, 3.69)	0.429	1.34 (0.56, 3.22)	0.512	0.78 (0.28, 2.16)	0.629
			n=431		n=431		n=432		n=431	

**Table 4:** Predictors of cycling on the path

Variable	Unadjusted		Maximally adjusted		Adjusted model without interaction effects		Sensitivity analysis: use not coded forward	
	OR (95% CI)	p	OR (95% CI)	p	OR (95% CI)	p	OR (95% CI)	p
Intervention								
Proximity to path (negative square root of distance in km)	**2.30 (1.88, 2.83)**	**<0.001**	**2.18 (1.58, 3.00)**	**<0.001**	**2.78 (2.11, 3.66)**	**<0.001**	**2.84 (2.13, 3.78)**	**<0.001**
Change in commute distance (km)	0.60 (0.22, 1.65)	0.324						
Socio-economics								
Gender								
Male	1.00		1.00		1.00		1.00	
Female	**0.48 (0.31, 0.73)**	**0.001**	**0.41 (0.24, 0.69)**	**0.001**	**0.42 (0.25, 0.70)**	**0.001**	**0.36 (0.21, 0.61)**	**<0.001**
Age								
≤30	0.52 (0.18, 1.45)	0.210	0.77 (0.25, 2.40)	0.658	0.76 (0.24, 2.34)	0.630	0.99 (0.31, 3.11)	0.983
31–40	1.00		1.00		1.00		1.00	
41–50	0.85 (0.50, 1.45)	0.558	0.84 (0.44, 1.58)	0.587	0.85 (0.45, 1.60)	0.622	0.87 (0.45, 1.65)	0.659
51–60	0.82 (0.49, 1.37)	0.450	0.83 (0.44, 1.54)	0.546	0.83 (0.45, 1.55)	0.564	0.81 (0.43, 1.53)	0.523
61+	0.60 (0.31, 1.17)	0.134	0.45 (0.20, 1.04)	0.061	0.47 (0.21, 1.07)	0.071	**0.38 (0.16, 0.90)**	**0.027**
Age								
Years	0.99 (0.98, 1.01)	0.372						
Education level								
No degree	1.00		1.00		1.00		1.00	
Degree	**1.63 (1.08, 2.45)**	**0.019**	1.06 (0.65, 1.74)	0.809	1.07 (0.66, 1.75)	0.779	1.32 (0.80, 2.18)	0.272
Car ownership								
No	1.00		1.00		1.00		1.00	
1 car	0.86 (0.45, 1.64)	0.644	0.87 (0.40, 1.89)	0.724	0.88 (0.40, 1.94)	0.756	0.84 (0.38, 1.87)	0.677
2 or more	0.56 (0.29, 1.09)	0.087	0.89 (0.39, 2.03)	0.776	0.89 (0.39, 2.06)	0.793	0.89 (0.38, 2.07)	0.784
Housing tenure								
Not owner	1.00							
Owner	1.25 (0.75, 2.09)	0.388						
Driving licence								
No	1.00							
Yes	1.49 (0.73, 3.03)	0.276						
Access to bicycle								
No								
Yes	a							
Student								
No	1.00							
Yes	0.90 (0.21, 3.81)	0.886						
Child(ren) in household								
No	1.00		1.00		1.00		1.00	
Yes	1.37 (0.90, 2.08)	0.140	1.07 (0.61, 1.85)	0.820	1.07 (0.62, 1.85)	0.802	1.24 (0.71, 2.14)	0.452
Health characteristics								
Mental health								
(MCS-8)	1.00 (0.98, 1.03)	0.771						
Physical health								
(PCS-8)	1.03 (1.00, 1.06)	0.075	**1.04 (1.01, 1.08)**	**0.019**	**1.04 (1.01, 1.08)**	**0.019**	**1.06 (1.02, 1.09)**	**0.003**
Limiting health condition								
No	1							
Yes	0.73 (0.39, 1.34)	0.307						
Difficulty walking^b^								
No	1.00							
Yes	0.29 (0.06, 1.35)	0.116						
Spatial characteristics								
Type of settlement								
Urban	1.00		1.00		1.00		1.00	
Town & Fringe	0.89 (0.53, 1.49)	0.663	**2.10 (1.04, 4.21)**	**0.038**	**2.48 (1.24, 4.93)**	**0.010**	**2.67 (1.32, 5.37)**	**0.006**
Village, Hamlet & Isolated Dwellings	0.70 (0.42, 1.18)	0.185	**2.65 (1.21, 5.78)**	**0.015**	**2.56 (1.25, 5.28)**	**0.011**	**2.93 (1.39, 6.21)**	**0.005**
Work characteristics								
Car parking at work								
No	1.00							
Yes, paid	0.88 (0.53, 1.47)	0.627						
Yes, free	0.86 (0.54, 1.36)	0.521						
Commute distance (km)								
	**0.92 (0.90, 0.95)**	**<0.001**						
Interaction variables								
Proximity *Town & Fringe			1.50 (0.79, 2.84)	0.220				
Proximity * Village, Hamlet & Isolated Dwellings			2.06 (1.00, 4.26)	0.051				
Constant			0.77 (0.10, 6.19)	0.808	1.09 (0.14, 8.37)	0.932	0.44 (0.05, 3.68)	0.45
			n=428		n=428		n=428	

a all users had a bicycle available

b not included: small n

## Appendix 1 Calculation of exposure and other distance measures

The exposure measures were calculated using geographic information system (GIS) software ArcGIS 9.3 and three created route networks: a car network (1) and two pedestrian and cyclist networks, reflecting the routes available before (2) and after (3) the intervention. These networks were created using the following data sources:

Car network: the Ordnance Survey (OS) MasterMap^®^ Integrated Transport Network (ITN) (Ordnance Survey (OS), 2013) road network dataset was used, minus the busway itself which is not available for use by private motor vehicles and was therefore removed manually

Pre-intervention pedestrian and cyclist network: motorways and the busway path were removed from the car network (1), and footpaths and cycle routes were added from local authority data on rights-of-way (public footpaths, bridleways and byways) (Cambridgeshire County Council, 2010), cycle route information from the UK charity Sustrans (2012) and any additional paths from OpenStreetMap.com (OpenStreetMap, 2010).

Post-intervention pedestrian and cyclist network: as the pre-intervention network (2) but with the addition of the busway path.

Home and workplace postcodes reported in the questionnaire were georeferenced to a point on the ground using the OS CodePoint^®^ database (Ordnance Survey (OS), 2012). Distances between participants׳ home and work locations before and after the intervention were calculated using the Network Analyst Route tool in ArcGIS and the pre- and post-intervention route networks. Distances between home and the nearest busway stop and the nearest access point to the busway path were calculated using the Network Analyst Closest Facility tool in ArcGIS, using the post-intervention pedestrian and cyclist network. Commute times by bus before and after the intervention were computed using Accession (Basemap, 2013), a software package which uses road networks, road speed data and public transport timetable information to calculate travel times. This was used to estimate the quickest potential journey to work on a Wednesday, with the journey commencing after 6am and the work destination to be reached before 10am – a four hour period chosen to represent the peak morning commute.

The exposure data were cleaned to remove changes of <5 m and merged with the socioeconomic and spatial data from the questionnaire. This allowed us to describe the characteristics of the sample and the sociospatial distribution of the exposure measures, and to confirm the choice of primary and secondary exposure measures while blinded to their relationship with the outcome measures. The data were then fully merged for the main analyses.

## Appendix 2

See Table B1.
